# Effects of Different Modified Biochars on Growth of *Kosteletzkya virginica* and Corresponding Transcriptome Analysis

**DOI:** 10.3390/plants14121849

**Published:** 2025-06-16

**Authors:** Hao Dai, Mingyun Jia, Jianhui Xue, Yuying Huang, Jinping Yu

**Affiliations:** 1 Jiangsu Key Laboratory for the Research and Utilization of Plant Resources, Institute of Botany, Jiangsu Province and Chinese Academy of Sciences (Nanjing Botanical Garden Mem. Sun Yat-Sen), Nanjing 210014, China; hdai@njfu.edu.cn (H.D.); jiamingyun@jib.ac.cn (M.J.); jhxue@njfu.edu.cn (J.X.); 2 College of Biomedicine and Health, Anhui Science and Technology University, Chuzhou 233100, China; 3 College of Ecology and Environment, Nanjing Forestry University, Nanjing 210037, China

**Keywords:** modified biochar, *Kosteletzkya virginica*, salt–alkali tolerance, transcription factor network, phytohormone signaling

## Abstract

Modified biochar can effectively improve the quality and environment of coastal saline–alkali soil, but its effects on the growth and development of halophytes and its mechanism are still unclear. This study systematically evaluated the growth-promoting effects and preliminary mechanisms of H_3_PO_4_-modified biochar (HBC) and H_3_PO_4_–kaolinite–biochar composite (HBCK) on the economically important halophyte *Kosteletzkya virginica*. The results demonstrated that the application of HBC/HBCK significantly enhanced plant growth, resulting in increases of over 55% in plant height and greater than 100% in biomass relative to the control. Multidimensional mechanistic analysis revealed the following: (1) accumulation of nitrogen (N), phosphorus (P), and potassium (K) increased by at least 40%, significantly enhancing nutrient uptake; (2) increases in the activities of superoxide dismutase (SOD) and peroxidase (POD) by over 100% and 70%, respectively, markedly boosting antioxidant capacity and effectively alleviating oxidative stress; (3) molecular regulation via the activation of transcription factor networks (HSP, MYB, TCP, AP2/ERF, bZIP, and NLP) and modulation of key genes in ABA, BR, and JA signaling pathways (CYP707A, CYP90, and OPR2), establishing a multi-layered stress adaptation and growth promotion system. Beyond assessing the growth-promoting effects of modified biochars, this study provides novel insights into the regulatory transcription factor networks and phytohormone signaling pathways, offering theoretical foundations for the molecular design of biochars for saline–alkali soil remediation.

## 1. Introduction

Coastal saline–alkali soils represent a distinct category of salt-affected soils primarily distributed in coastal regions. Their formation is closely associated with seawater intrusion, high mineralization of groundwater, and anthropogenic activities [1]. As one of China’s predominant saline soil types, coastal saline–alkali soils cover an area of approximately 7 × 10^6^ hm^2^, predominantly in economically developed southeastern coastal zones, presenting significant reclamation potential [2]. In recent years, advancements in land reclamation techniques have facilitated the conversion of coastal tidal flats into arable land, alleviating land-use conflicts [3]. However, the inherently poor soil quality and restricted crop productivity in coastal saline–alkali soils severely impair land-use efficiency and agricultural yield potential [4,5]. Given China’s shrinking arable land resources and growing food security concerns, the rational utilization of coastal saline–alkali soils holds strategic importance in balancing land demand and ensuring food security [6,7]. Traditional remediation methods, including freshwater leaching, gypsum application, and soil replacement, face constraints in coastal regions due to freshwater scarcity, high costs, and secondary pollution risks [8,9]. Thus, there is an urgent need to develop efficient and sustainable remediation strategies.

Biochar, a porous carbonaceous material produced through pyrolysis of organic biomass under oxygen-limited conditions, exhibits remarkable potential in improving soil physicochemical properties, enhancing nutrient availability, and promoting carbon sequestration due to its high surface area, abundant functional groups, and stable carbon structure [10,11,12]. It has been widely applied in the remediation of degraded soils with promising results. For instance, research demonstrated that biochar effectively increases soil pH and nutrient utilization in acidic soils [13]. However, its application in saline–alkali soils remains controversial. Since most biochars are inherently alkaline and rich in basic cations [14], direct application may exacerbate soil conditions. For example, studies have reported that the direct application of biochar can inhibit crop growth and reduce crop yield [15,16]. To address these limitations, recent research has focused on modifying biochar to enhance its functionality [17]. For instance, acid modification and mineral modification increased the cation exchange capacity (CEC) of biochar by 44.8% and 48.5%, respectively. Modifications involving soil minerals, metal oxides, and alkaline agents resulted in increases of 20.9%, 15.7%, and 13.6% in ash content. Furthermore, acidic and H_2_O_2_ modifications yielded the highest specific surface area (SSA) enhancements of 57.1% and 53.4%. To develop modified biochar with potential for application in coastal saline–alkaline soils, this study employed phosphoric acid and mineral loading for modification. Phosphoric acid modification significantly increased the SSA and porosity of the biochar while introducing acidic functional groups, lowering its pH, and enhancing its adsorption capacity for metal ions [18]. Mineral-loaded modifications (e.g., with montmorillonite or kaolinite) not only improved biochar stability but also augmented its SSA and adsorption capacity [19]. By retaining the inherent advantages of the porous biochar structure, these modifications successfully imparted more suitable acid–base properties and stronger adsorption performance, resulting in an ameliorant with remediation potential for coastal saline–alkaline soils. However, its specific mechanisms of action on halophytes remain unclear. *Kosteletzkya virginica* holds significant value in saline soil remediation due to its unique salt-tolerance mechanisms [20]. As an economically important halophyte, it serves multiple purposes, including oil production, papermaking, medicinal uses, ornamental applications, and ecological restoration, while simultaneously improving soil aeration, water permeability, and organic matter content in coastal saline environments [21,22]. Despite its adaptability, high salinity and low nutrient availability in saline–alkali soils still constrain its growth performance. Consequently, enhancing its stress resistance and growth efficiency through exogenous amendments has become a key research focus.

In this study, the effects of modified biochar on the growth of *K. virginica* were systematically studied in a pot experiment to visually evaluate the improvement effect. Combined with transcriptome sequencing technology, the mechanism of modified biochar was preliminarily analyzed from the perspectives of nutrient absorption, the antioxidant system, and molecular regulation. Based on our findings, we propose the following scientific hypothesis: HBC and HBCK enhance the development of *K. virginica* through the following: (1) augmented root-mediated nutrient acquisition, (2) elevated antioxidant enzyme activity mitigating saline–alkali stress, and (3) reprogrammed physiological metabolism and signaling transduction via coordinated multi-gene regulatory networks. This multi-scale approach deciphers the integrated soil–biochar–plant continuum driving coastal saline soil rehabilitation, bridging material science and plant molecular ecology to advance sustainable land restoration strategies.

## 2. Results

### 2.1. Effects of Different Modified Biochars on the Growth Parameters of K. virginica

**Figure 1:**
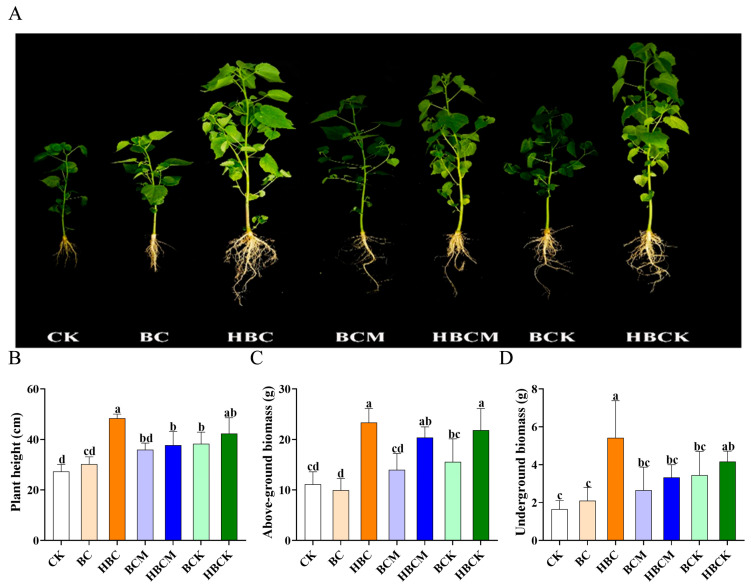
The effects of different modified biochar applications on the growth indicators of *K. virginica*. (**A**). Growth morphology of *K. virginica* after the application of different modified biochars. (**B**). Statistical chart of the plant height of *K. virginica* after the application of different modified biochars. (**C**). Statistical chart of the aboveground biomass of *K. virginica* after the application of different modified biochars. (**D**). Statistical chart of the underground biomass of *K. virginica* after the application of different modified biochars; different small letters in each column indicate significant differences (*p* < 0.05) among biochars and modified biochar composites. (CK: control group; BC: raw corn straw biochar; HBC: H_3_PO_4_-modified biochar; BCM: montmorillonite–biochar composite; HBCM: H_3_PO_4_–montmorillonite–biochar composite; BCK: kaolinite–biochar composite; HBCK: H_3_PO_4_–kaolinite–biochar composite.).

To evaluate the growth-promoting effects of modified biochar on *K. virginica*, this study investigated the impacts of different modified biochar treatments on plant height, aboveground biomass, and underground biomass. The results demonstrated that different modified biochars exerted distinct effects on plant morphogenesis (Figure 1A). Regarding plant height, the BC (raw corn straw biochar) treatment showed no significant effect, whereas HBC (H_3_PO_4_-modified biochar), BCM (montmorillonite–biochar composite), HBCM (H_3_PO_4_–montmorillonite–biochar composite), BCK (kaolinite–biochar composite), and HBCK (H_3_PO_4_–kaolinite–biochar composite) treatments significantly increased plant height by 77.5%, 31.8%, 38.5%, 40.6%, and 55.1%, respectively. Notably, HBC and HBCK treatments exhibited the most pronounced effects on plant height enhancement (Figure 1B). These findings suggest that phosphoric acid modification alone or in combination with kaolin significantly enhances biochar’s ability to promote vertical plant growth. In terms of biomass, the BC treatment did not significantly improve either aboveground or underground biomass. In contrast, the HBC and HBCK treatments demonstrated substantial growth-promoting effects, with HBC increasing aboveground and underground biomass by 1.1-fold and 2.3-fold, respectively, and HBCK enhancing them by 1.0-fold and 1.5-fold, respectively. Although BCM, HBCM, and HBK also exhibited positive effects, the improvements (0.3–1.1-fold) were less pronounced than those of HBC and HBCK (Figure 1C,D).

In summary, the results indicate that BC had no significant effect on root growth promotion, and single mineral modification BCM showed limited efficacy. However, the HBC and HBCK treatments consistently exhibited the most robust growth-promoting effects across all three parameters: plant height, aboveground biomass, and underground biomass.

### 2.2. Analysis of Nutrient Element Indicators in K. virginica with Different Modified Biochars

**Figure 2:**
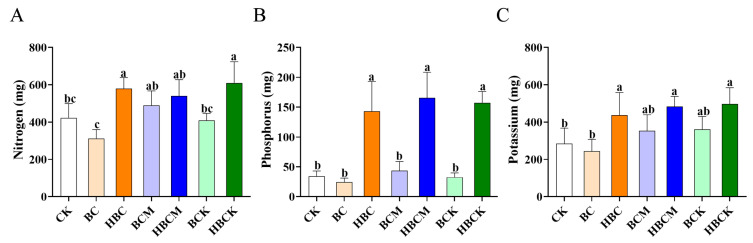
The effects of different modified biochar applications on the nutrition indicators of *K. virginica*. (**A**). The nitrogen content of *K. virginica* after the application of different modified biochars. (**B**). The phosphorus content of *K. virginica* after the application of different modified biochars. (**C**). The potassium content of *K. virginica* after the application of different modified biochars; different small letters in each column indicate significant differences among biochars and modified biochar composites; *p* < 0.05. (CK: control group; BC: raw corn straw biochar; HBC: H_3_PO_4_-modified biochar; BCM: montmorillonite–biochar composite; HBCM: H_3_PO_4_–montmorillonite–biochar composite; BCK: kaolinite–biochar composite; HBCK: H_3_PO_4_–kaolinite–biochar composite.).

To assess the effects of modified biochars on nutrient uptake in *K. virginica*, we measured nutritional indices under various biochar treatments. The results demonstrated that modified biochar application significantly influenced the plant’s enrichment capacity for nitrogen (N), phosphorus (P), and potassium (K). Regarding nitrogen absorption, the total N content in the HBC-, HBCK-, and BCM-treated groups increased significantly by 0.4-fold, 0.4-fold, and 0.2-fold, respectively, compared with the control (CK). In contrast, the BC-, BCM-, and BCK-treated groups showed no significant differences from CK (Figure 2A). For phosphorus accumulation, the HBC, HBCM, and HBCK treatments exhibited remarkable enrichment effects, enhancing P content by 3.2-, 3.8-, and 3.6-fold, respectively, relative to CK. However, the BC, BCM, and BCK treatments had no significant impact on P levels (Figure 2B), indicating that phosphate group incorporation is crucial for improving biochar’s phosphorus-activating capacity. In terms of potassium accumulation, the HBC, HBCM, and HBCK treatments showed the most pronounced effects, increasing the K content by 0.5-, 0.7-, and 0.7-fold, respectively, compared with CK. Other treatment groups displayed no significant differences from CK (Figure 2C).

In summary, both the HBC and HBCK modification methods significantly promoted the enrichment of all three major nutrient elements (N, P, and K), suggesting that these modified biochars may enhance *K. virginica* growth through improved nutrient accumulation. However, the underlying molecular mechanisms require further investigation.

### 2.3. Impacts of Different Modified Biochars on Antioxidant Enzyme Activity in K. virginica

**Figure 3:**
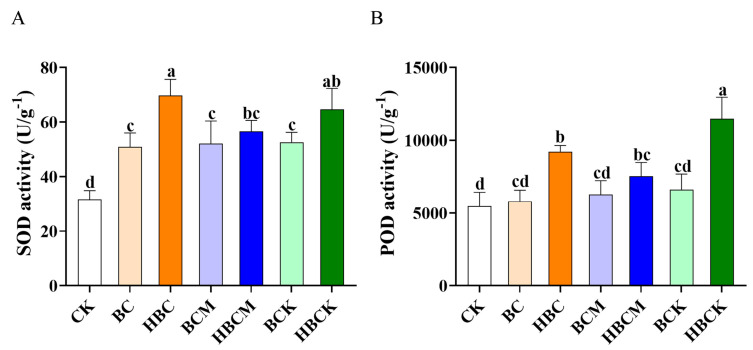
The effects of different modified biochar applications on the antioxidant enzyme activity of *K. virginica*. (**A**). The effects of applying different modified biochars on the SOD activity of *K. virginica*. (**B**). The effects of applying different modified biochars on the POD activity of *K. virginica*. Different small letters in each column indicate significant differences among biochars and modified biochar composites; *p* < 0.05. (CK: control group; BC: raw corn straw biochar; HBC: H_3_PO_4_-modified biochar; BCM: montmorillonite–biochar composite; HBCM: H_3_PO_4_–montmorillonite–biochar composite; BCK: kaolinite–biochar composite; HBCK: H_3_PO_4_–kaolinite–biochar composite.).

To investigate whether biochar enhances saline–alkali stress resistance in *K. virginica* by regulating antioxidant enzyme activity, we measured the effects of different treatments on superoxide dismutase (SOD) and peroxidase (POD) activities. As shown in Figure 3A, all biochar treatments significantly increased the SOD activity, with HBC and HBCK exhibiting the most pronounced effects, elevating the SOD activity by 1.2-fold and 1.1-fold, respectively. For POD activity, only the HBC, HBCM, and HBCK treatments showed significant enhancements, increasing the POD levels by 0.7-fold, 0.4-fold, and 1.1-fold, respectively. Among these, the HBC and HBCK treatments demonstrated superior induction effects compared with the other groups (Figure 3B).

The results indicate that the HBC and HBCK treatments were the most effective in activating both the SOD and POD activities. Previous studies have confirmed a positive correlation between elevated SOD/POD activities and plant biomass accumulation [23]. Our findings suggest that the HBC and HBCK treatments significantly enhance antioxidant enzyme activities, effectively scavenging reactive oxygen species (ROSs) induced by saline–alkali stress. This mechanism helps maintain cell membrane integrity and photosynthetic function, thereby improving *K. virginica*’s growth adaptability in saline–alkali environments.

### 2.4. Quality Analysis of the Transcriptome Data of K. virginica Roots

To elucidate the molecular mechanisms by which HBC and HBCK modified biochars enhance the growth of *K. virginica* in saline–alkali soil, we performed a comparative transcriptomic analysis of root tissues under different biochar treatments. Total RNA was extracted from four experimental groups: CK, BC, HBC, and HBCK. Quality assessment showed that all RNA samples met stringent criteria for subsequent sequencing, with concentrations of >300 ng/μL, A260/A280 ratios between 2.0–2.2 (Table S1), and clear 28S/18S/5S ribosomal bands on 1.2% agarose gels (Figure S1A), indicating high-quality, intact RNA suitable for library construction. Sequencing of 12 samples generated 84.41 Gb of clean data, with each sample yielding ≥ 6.13 Gb. After rigorous quality control, including adapter trimming and low-quality read removal, all samples exhibited excellent base quality scores of Q20 > 98.7% and Q30 >95.99% (Table 1), confirming the reliability of the data for downstream analysis. Transcript assembly revealed a characteristic length distribution of unigenes (200–3000 bp), with the majority (65%) falling within the 200–1000 bp range (Figure S1B), which was consistent with the expected eukaryotic transcript sizes and demonstrated proper library preparation. These high-quality transcriptomic data provide a solid foundation for subsequent differential expression.

### 2.5. Gene Function Annotation Analysis and Differential Gene Statistics

Functional annotation of unigenes using BLAST+ 2.15.0 against multiple databases (Nr, Swiss-Prot, Pfam, eggNOG, GO, KEGG) identified 44,137 (60.21%) annotated genes (Table 2). The Nr database showed the highest annotation rate (43,828; 59.79%), indicating strong homology with known species. The GO analysis annotated 37,354 genes (50.96%) across the molecular function, biological process and cellular component categories. Additional annotations included eggNOG (34,955; 47.69%), Swiss-Prot (32,555; 44.41%), Pfam (28,317; 38.63%), and KEGG (18,529; 25.28%) pathways, primarily in metabolic and signal transduction pathways. This comprehensive annotation provides a foundation for subsequent differential gene expression analysis.

**Figure 4:**
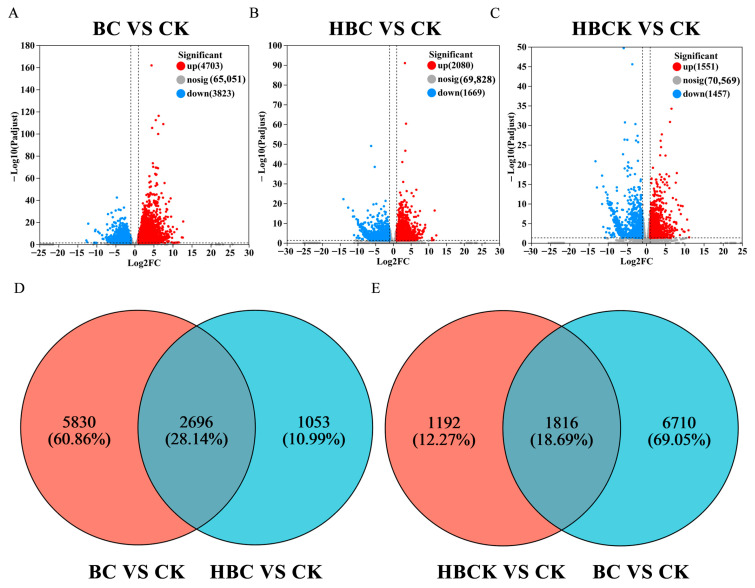
Volcanic and Venn diagram analysis of differential gene numbers. (**A**). Volcanic map of differential genes between BC and CK. (**B**). Volcanic map of differential genes between HBC and CK. (**C**). Volcanic map of differential genes between HBCK and CK. (**D**). Venn plots of BC vs. CK and HBC vs. CK. (**E**). Venn plots of BC vs. CK and HBCK vs. CK. (CK: control group; BC: raw corn straw biochar; HBC: H_3_PO_4_-modified biochar; HBCK: H_3_PO_4_–kaolinite–biochar composite.).

Applying stringent statistical thresholds (|log2 (Fold Change)| ≥ 1, FDR < 0.05), we conducted comprehensive transcriptome analysis to characterize treatment-induced gene expression changes. The volcano plot analysis demonstrated distinct gene expression patterns across treatment groups; compared with the CK treatment, the BC treatment induced substantial transcriptional changes, with 8526 DEGs identified (4703 upregulated and 3823 downregulated), demonstrating its broad but nonspecific regulatory effects (Figure 4A). Notably, the HBC treatment resulted in 3749 DEGs relative to CK (2080 upregulated, 1669 downregulated; Figure 4B), while the HBCK treatment generated 3008 DEGs (1551 upregulated, 1457 downregulated; Figure 4C). To isolate modification-specific effects, we performed a Venn analysis to identify unique DEG sets. This revealed 1053 genes specifically activated by HBC (Figure 4D) and 1192 genes uniquely regulated by HBCK (Figure 4E), providing targeted gene pools for subsequent functional characterization.

### 2.6. GO Enrichment Classification of Differentially Expressed Genes

**Figure 5:**
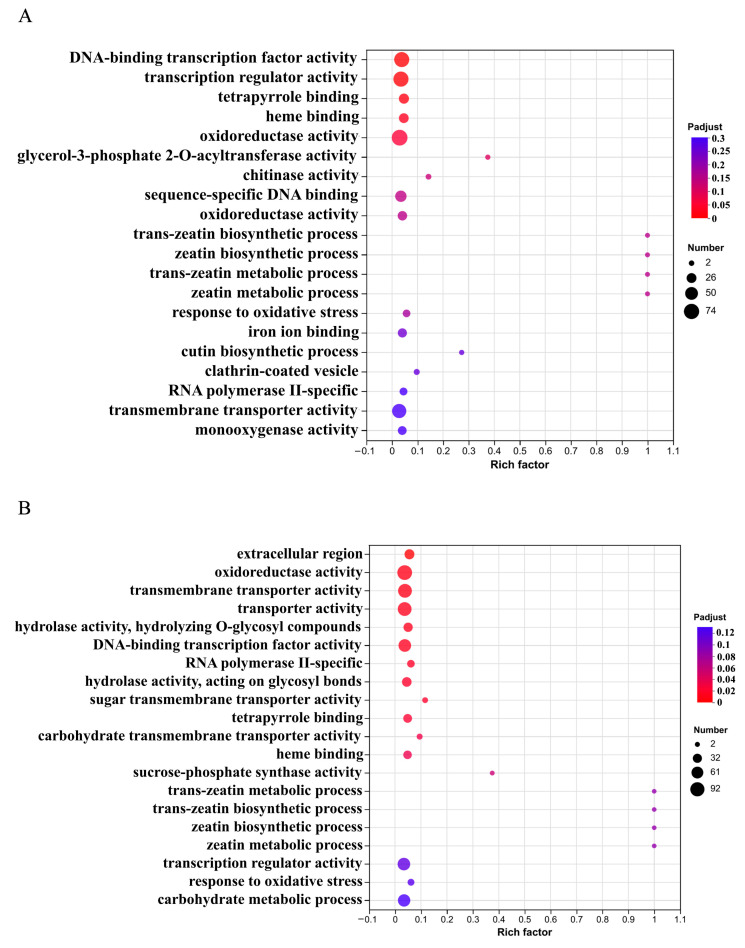
GO enrichment classification analysis of differentially expressed genes. (**A**). GO enrichment classification analysis of differentially expressed genes treated with HBC. (**B**). GO enrichment classification analysis of differentially expressed genes treated with HBCK. (HBC: H_3_PO_4_-modified biochar; HBCK: H_3_PO_4_–kaolinite–biochar composite.).

GO enrichment analysis of differentially expressed genes (DEGs) revealed that the HBC-treated samples showed significant enrichment in the molecular function category, with 67 genes annotated for “DNA-binding transcription factor activity” and 68 genes involved in “transcription regulator activity” (Figure 5A), suggesting that HBC enhances stress signal transduction by remodeling transcriptional regulatory networks. Additionally, 74 genes were enriched for “oxidoreductase activity”, indicating that biochar may improve ROS scavenging capacity and maintain redox homeostasis by modulating oxidoreductase activities, thereby enhancing *K. virginica*’s adaptation to saline–alkali stress. Similarly, DEGs from the HBCK treatment were predominantly enriched in molecular function terms, with 92 genes associated with “oxidoreductase activity” and 66 genes participating in “transcription regulator activity”, further confirming the crucial roles of oxidoreductases and transcription factors in HBCK-mediated saline–alkali stress alleviation and growth promotion. Notably, 80 genes were linked to “transmembrane transporter activity” and “transporter activity” (Figure 5B), suggesting that HBCK may additionally mitigate saline–alkali stress by regulating ion transmembrane transport to maintain cellular osmotic balance.

**Figure 6:**
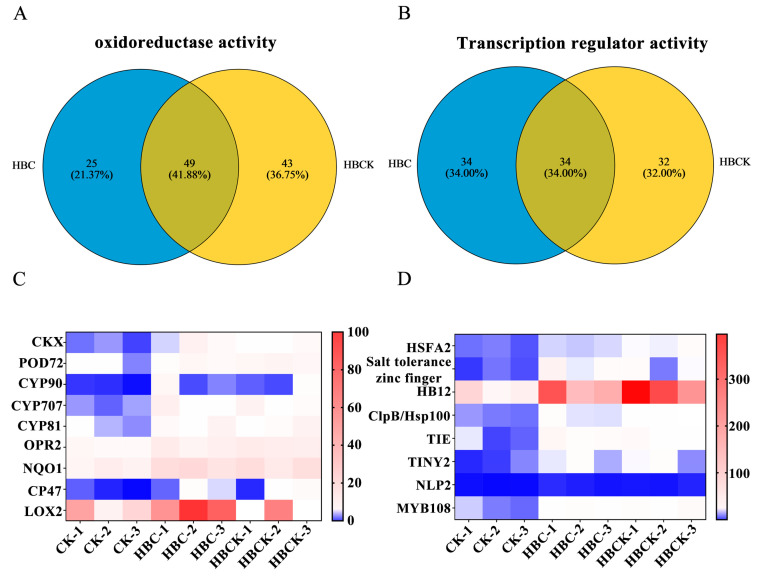
Differential gene analysis of the co-enrichment of HBC and HBCK. (**A**). Venn plots of oxidoreductase-activity-related genes co-enriched by HBC and HBCK. (**B**). Venn plots of transcriptional-regulation-activity-related genes co-enriched by HBC and HBCK. (**C**). Heat map of the expression of oxidoreductase genes co-enriched with HBC and HBCK. (**D**). Heat map of the expression of transcription factor genes co-enriched by HBC and HBCK. (CK: control group; HBC: H_3_PO_4_-modified biochar; HBCK: H_3_PO_4_–kaolinite–biochar composite; *CKX:* cytokinin oxidase/dehydrogenase; *POD72*: peroxidase 72; *CYP*: cytochrome P450 genes; *OPR2*: 12-oxophytodienoate reductase 2; *NQO1*: NAD(P)H: quinone oxidoreductase 1; *CP47*: photosystem II CP47 reaction center protein gene; *LOX2*: lipoxygenase 2; *HSFA2*: heat shock transcription factor A2; *HB12*: homeodomain-leucine zipper class I, *TIE:* TCP transcription factor; *TINY2*: AP2/ERF transcription factor; *NLP2:* NLP transcription factor; *MYB108*: MYB transcription factor.).

GO analysis revealed that the differentially expressed genes (DEGs) induced by both the HBC and HBCK treatments were significantly enriched in “oxidoreductase activity” and “transcription regulator activity”. To further identify the specific genes involved, we analyzed the common redox-related enzymes and transcription factors enriched in both treatments. As shown in Figure 6, there were 49 commonly enriched redox-related genes (Figure 6A) and 34 transcription factors (Figure 6B). Expression analysis of genes previously reported to participate in abiotic stress responses and growth regulation showed significant upregulation of these genes. The upregulated oxidoreductase-related genes included cytokinin oxidase/dehydrogenase (*CKX*), peroxidase 72 (*POD72*), cytochrome P450 genes (*CYP90*, *CYP707*, *CYP81*), 12-oxophytodienoate reductase 2 (*OPR2*), NAD(P)H: quinone oxidoreductase 1 (*NQO1*), photosystem II CP47 reaction center protein gene (*CP47*), and lipoxygenase 2 (*LOX2*). These genes contribute to plant adaptation to saline–alkali stress and growth regulation through their involvement in the biosynthesis and metabolism of various phytohormones (cytokinins, brassinosteroids, abscisic acid, jasmonic acid) and related metabolites (phenylpropanoids, fatty acids, flavonoids), as well as through their effects on photosystem II (Figure 6C). The transcription regulatory activity was primarily mediated by several transcription factors, including heat shock transcription factor A2 (*HSFA2*), salt tolerance zinc finger transcription factor, homeodomain-leucine zipper class I (*HB12*), *ClpB/Hsp100*, TCP transcription factor *TIE*, AP2/ERF transcription factor *TINY2*, NLP transcription factor *NLP2*, and MYB transcription factor *MYB108* (Figure 6D). These transcription factors participate in abiotic stress responses and plant growth and development processes by regulating specific gene activities.

### 2.7. KEGG Enrichment Analysis of Differentially Expressed Genes

**Figure 7:**
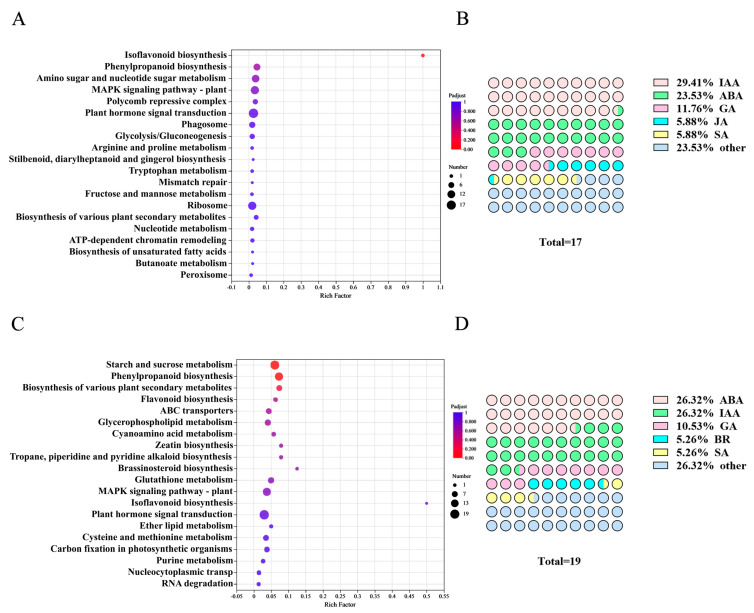
KEGG enrichment analysis of differentially expressed genes. (**A**). KEGG enrichment analysis of differentially expressed genes treated with HBC. (**B**). Waffle diagram of plant hormone-signal-transduction-related genes induced by HBC. (**C**). KEGG classification analysis of differentially expressed genes treated with HBCK. (**D**). Waffle diagram of plant hormone-signal-transduction-related genes induced by HBCK. (HBC: H_3_PO_4_-modified biochar; HBCK: H_3_PO_4_–kaolinite–biochar composite; IAA: auxin; ABA: abscisic acid; GA: gibberellin; JA: jasmonic acid; SA: salicylic acid; BR: brassinosteroid).

KEGG pathway enrichment analysis revealed that both the HBC and HBCK treatments significantly altered the gene expression profiles in *K. virginica* roots, with differentially expressed genes (DEGs) being predominantly enriched in key metabolic pathways including “plant hormone signal transduction”, “MAPK signaling pathway”, “starch and sucrose metabolism”, and “phenylpropanoid biosynthesis”. These pathways have been well documented to play crucial roles in regulating plant growth and development, suggesting that these modified biochars may function as exogenous regulators by modulating the expression of key enzymes and related genes in these metabolic pathways to promote *K. virginica* growth. Notably, among all of the enrichment pathways, the number of differential “plant hormone signal transduction” genes were the largest. Specifically, HBC treatment induced significant expression changes in 17 genes (Figure 7A), which were primarily involved in auxin (IAA), abscisic acid (ABA), gibberellin (GA), jasmonic acid (JA) and salicylic acid (SA) signaling pathways, among which IAA-signaling-related genes were the most abundant (29.41%). In contrast, the HBCK treatment resulted in the differential expression of 19 genes (Figure 7B), which were mainly associated with the auxin, ABA, GA, SA, and brassinosteroid (BR) signaling pathways, with IAA- and ABA-related genes being the most numerous (26.32%). These findings strongly demonstrate that both HBC- and HBCK-modified biochars likely exert significant effects on plant growth and development through specific regulation of key genes in plant hormone signaling pathways in *K. virginica* roots. Particularly, the distinct gene regulatory patterns observed in hormone signaling pathways between different treatments provide important clues for understanding the molecular mechanisms underlying plant growth promotion by modified biochars.

### 2.8. RT-qPCR Analysis

**Figure 8:**
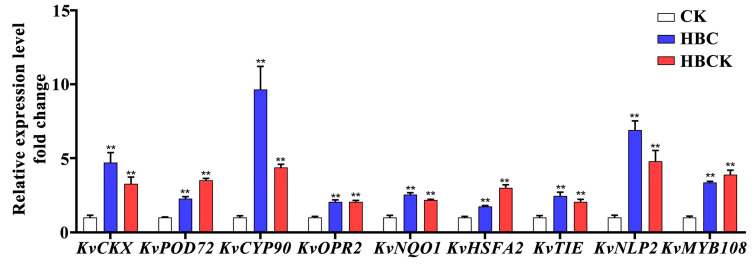
RT-qPCR analysis of differentially expressed genes. ** in each column indicates significant differences among CK and modified biochar composites (HBC and HCBK); *p* < 0.01. (CK: control group; HBC: H_3_PO_4_-modified biochar; HBCK: H_3_PO_4_–kaolinite–biochar composite.).

To validate the transcriptome sequencing results, we selected nine genes related to redox regulation and transcriptional activity for RT-qPCR analysis. The results demonstrated significant upregulation of these genes under both the HBC and HBCK treatments (Figure 8). Specifically, the expression levels of *KvCKX*, *KvPOD72*, *KvCYP90*, *KvOPR2*, *KvNQO1*, *KvHSFA2*, *KvTIE*, *KvNLP*, and *KvMYB108* were increased by average factors of 3.99, 2.89, 7.02, 2.06, 2.36, 2.38, 2.26, 5.84, and 3.63 under the HBC treatment, respectively. Correspondingly, the HBCK treatment resulted in fold changes of 3.27, 3.51, 4.39, 2.06, 2.18, 3.01, 2.06, 4.79, and 3.89 for these genes, respectively. The expression patterns observed using RT-qPCR were consistent with the transcriptome sequencing data, confirming the reliability of our sequencing results. These findings provide compelling evidence for the involvement of these genes in enhancing *K. virginica*’s adaptation to saline–alkali stress and promoting plant growth under the HBC and HBCK treatments. Furthermore, this study establishes a theoretical foundation for future investigations of the mechanisms of biochar-mediated plant growth promotion in saline soils and facilitates the identification of salt-tolerance genes in *K. virginica*.

## 3. Discussion

### 3.1. Effects of Different Modified Biochars on K. virginica Growth and Development

Studies have demonstrated that modified biochars significantly enhance plant salt tolerance by optimizing soil porosity, adsorbing salt ions, and regulating microbial activity. For instance, one study innovatively applied a combined strategy of maize straw biochar–zinc oxide nanocomposites (MB-ZnO) and *Trichoderma harzianum*-loaded biochar (MBT) in a *Sesbania sesban* cultivation trial within Cd-Cu co-contaminated soil. This synergistic approach effectively immobilized soil heavy metals while significantly enhancing plant physiological and biochemical parameters. Specifically, the root length and plant height of *S. sesban* increased by 30% and 33.33%, respectively, with a 60% improvement in biomass [24]. In the present study, treatments with modified biochar (HBC and HBCK) markedly promoted the growth and development of *K. virginica* seedlings under saline–alkaline stress conditions. The plant height increased by >50%, and the biomass was enhanced by 100% (Figure 1). These results demonstrate the significant efficacy of the prepared biochars in mitigating saline–alkaline stress and stimulating plant growth. Furthermore, they highlight the necessity of tailoring modified biochar designs to specific soil environments and stress factors to address agricultural requirements.

### 3.2. Effects of Modified Biochars on Nutrient Uptake in K. virginica Roots

Nitrogen (N), phosphorus (P), and potassium (K) play vital roles in maintaining plant nutrient homeostasis, promoting carbon assimilation, and supporting growth [25]. Dynamic changes in nutrient absorption are central to understanding biochar-mediated yield improvements [26]. Biederman et al. [27] summarized the published literature and found that biochar application generally enhances K uptake but shows inconsistent effects on N and P absorption across studies, a trend corroborated by domestic research. For example, while biochar increased rapeseed yield in pot experiments, it reduced N and P concentrations in shoots while elevating K levels [28]. Notably, some studies reported enhanced N uptake, reflected in higher total N accumulation at plant cultivation [29]. For P, Asai et al. revealed that the efficacy of biochar in modulating rice yield is critically dependent on soil phosphorus availability, demonstrating that biochar application exerts pronounced yield-enhancing effects specifically in phosphorus-deficient soil conditions [30]. These discrepancies likely stem from interactions among biochar ash composition, native soil nutrients, and crop metabolic traits, highlighting the complexity of nutrient regulation. Compared with these reported studies, the HBC- and HBCK-modified biochars prepared in this study did not promote the absorption of a single nutrient but significantly increased the absorption of multiple nutrients, showing a significant increase in N, P, and K and indicating their significant role in enhancing root nutrient uptake for plant growth.

### 3.3. HBC and HBCK Enhance Antioxidant Enzyme Activity and Salt–Alkali Stress Tolerance in K. virginica

The antioxidant enzyme system is critical for scavenging ROSs, regulating cellular osmotic potential, and protecting cells under stress. Biochar-based fertilizers can elevate antioxidant enzyme activity and root vitality while reducing ROS accumulation, thereby mitigating abiotic stress and promoting crop growth [31]. For instance, nitrate-modified biochar increased key antioxidant enzyme (SOD, CAT) activity and reduced hydrogen peroxide (H_2_O_2_) and malondialdehyde (MDA) levels in pakchoi grown in saline soil [32], while the HBC and HBCK treatments significantly boosted SOD and POD activities in *K. virginica* leaves (Figure 3), suggesting that different modified materials may have different effects on various antioxidant enzymes.

### 3.4. Molecular Response Mechanism of K. virginica to HBC and HBCK Application

Previous findings demonstrated that the application of HBC and HBCK effectively enhanced the growth of *K. virginica* in saline–alkali soil. Preliminary investigations suggested that modified biochar improves nutrient uptake in plant roots and enhances antioxidant enzyme activity, partially explaining the growth-promoting effects of biochar. To further elucidate the molecular mechanisms underlying *K. virginica*’s response to saline–alkali stress under biochar treatment, we conducted high-throughput sequencing to compare gene-level changes induced by HBC and HBCK. Transcriptomic analysis revealed a multidimensional regulatory mechanism by which HBC and HBCK modulate the salt stress response in *K. virginica*.

At the redox enzyme level, the expression of *CKX*, *POD72*, *CYP90*, *CYP707*, *CYP81*, *OPR2*, *NQO1*, *CP47*, and *LOX2* was significantly upregulated. Among these, CKX is a rate-limiting enzyme in the cytokinin degradation pathway and participates in plant salt stress responses [33,34]. For instance, overexpression of *MsCKX* enhanced salt stress tolerance in Arabidopsis by maintaining a high K^+^/Na^+^ ratio, boosting the activity of antioxidant enzymes to scavenge ROSs, and increasing the expression of stress-related genes (*P5CS1*, *DREB2*, ion transporters, and H^+^-pumps) [34]. Therefore, HBC and HBCK may promote cytokinin degradation by upregulating *CKX* gene expression, thereby suppressing its negative regulatory effects under high salt stress. The *POD72* gene encodes peroxidase (POD), a key enzyme for ROS scavenging under abiotic stress, playing a crucial role in plant growth, development, and stress responses [35]. The elevated expression of *POD72* corroborates the previously observed increase in POD enzyme activity, further suggesting that HBC and HBCK may alleviate salt–alkali stress damage in *K. virginica* by enhancing POD activity. The CYP90 family is responsible for brassinosteroid biosynthesis, acting as signaling factors in stress responses [36] and playing vital roles in plant growth and development. For example, ZmCYP90D1 in maize has been reported to regulate internode development by modulating brassinosteroid-mediated cell division and growth [37]. CYP707 is involved in ABA metabolism, regulating ABA levels under various stress conditions and participating in fruit ripening and abiotic stress responses [38]. Thus, the upregulated *CYP707A* in this study may exert important functions in plant growth and salt–alkali stress responses by influencing the ABA metabolic pathway. The CYP81 family genes have been reported to strongly respond to salt stress. For instance, *ABA* was shown to affect the ZAT12-mediated ROS signaling pathway in wheat, subsequently activating the expression of ROS-scavenging enzyme genes such as APX and CAT, reducing ROS accumulation and toxicity under salt stress, and conferring salt tolerance. Overexpression of *TaCYP81D5* in wheat improved salt tolerance during the seedling stage under hydroponic conditions [39]. Furthermore, a significant upregulation of CYP81 gene expression was observed, which likely contributes to the enhanced salt stress tolerance in K. virginica following modified biochar treatment. *OPR2* is a key gene in JA synthesis. JA is an important phytohormone that not only regulates plant growth and development but also serves as a signaling molecule in plant responses to biotic and abiotic stresses [40]. Concurrently, the observed upregulation of OPR2 gene expression suggests that the modified biochar treatments may influence phytohormone signaling pathways within the plant root system. NQO1 is a ubiquitous flavoprotein in eukaryotic cells that prevents oxidative DNA damage caused by environmental stressors. Additionally, *NQO1* plays a critical role in protecting endogenous antioxidants by maintaining the reduced forms of ubiquinone and α-tocopherol quinone [41]. Therefore, the upregulation of *NQO1* may contribute to mitigating salt–alkali-induced cellular damage. CP47 is a component of the PSII core complex. Studies have demonstrated that high salt stress severely damages the photosystem II reaction center in tomato, significantly reducing light-use efficiency and ultimately inhibiting plant growth [42]. Hence, HBC and HBCK may stabilize PSII and alleviate damage by modulating CP47, thereby reducing growth inhibition. *LOX* plays a pivotal role in plant defense responses to biotic and abiotic stresses [43]. For example, *BnaLOX3/4/6* in rapeseed was strongly upregulated under salt and drought stress and is an early-response gene to MeJA and SA [44]. Notably, a pronounced increase in lox2 gene expression was detected, potentially linked to the coordinated activation of multiple phytohormone signaling pathways in response to modified biochar application, which may further contribute to stress amelioration.

At the transcriptional regulation level, key transcription factors (TFs) such as *HSPA2*, *Zinc-Finger*, *MYB*, *TCP*, *AP2/ERF*, *bZIP*, and *NLP2* were significantly upregulated. These regulatory factors serve as central nodes in the salt stress response network, orchestrating downstream gene expression cascades to confer stress resistance. Heat shock proteins (HSPs) play a crucial role in plant responses to environmental stress. For instance, overexpression of wild barley *HvHSP16.9* enhanced salt tolerance in Arabidopsis, while its silencing significantly reduced salt tolerance in barley [45]. Zinc-finger proteins are also pivotal in salt stress responses. For example, overexpression of wheat *TaZNF* notably improved salt tolerance in transgenic Arabidopsis [46]. HB12, a homeodomain-leucine zipper (HD-Zip) TF, is widely reported to mediate responses to external stimuli and hormonal signals, regulating critical developmental processes such as root and stem elongation, leaf morphogenesis, inflorescence branching, and flowering [47]. In Arabidopsis, ATHB12 acts as a positive regulator of cell expansion during leaf development by upregulating genes like EXPANSIN A10 (*EXPA10*) and DWARF4 (*DWF4*) [48]. TIE2, an EAR motif-containing TF, interacts with TCP proteins to modulate leaf development. Heterologous overexpression of *GhTIE1* in cotton and Arabidopsis induced increased branching and altered plant morphology [49]. *KvNLP2* belongs to the NIN-like protein (NLP) family, a group of plant-specific TFs involved in nutrient uptake, growth, development, and stress responses [50]. In rice, OsNLP4 regulates genes associated with nitrogen absorption, assimilation, and signaling, playing a central role in nitrogen metabolism. *OsNLP4* mutants exhibit stunted seedling growth. MYB108, a member of the MYB TF family, mediates hormone signaling and developmental pathways. For example, Zhang et al. [51] demonstrated that MYB108 interacts with LBD29 to modulate auxin signaling, thereby regulating lateral root development. Consequently, HBC and HBCK treatments likely enhance the growth of *K. virginica* by upregulating multiple growth-related transcription factors that participate in diverse regulatory pathways. 

In terms of phytohormone metabolism, significant enrichment was observed in genes associated with IAA, ABA, and GA signaling pathways. These hormones play the following pivotal roles in plant development: (1) IAA serves as a crucial regulator of root growth and development, with its biosynthesis, transport, and metabolism directly or indirectly modulating root system architecture [52]; (2) ABA governs seed dormancy/germination, root growth, stomatal closure, and leaf senescence [53]; (3) GA functions as a growth promoter throughout the plant life cycle, influencing seed germination, stem elongation, and fruit development [54]. These findings demonstrate that HBC and HBCK can stimulate plant growth by modulating these critical hormone signaling pathways.

While previous studies have demonstrated the crucial roles of these key regulatory factors in plant responses to environmental stress and growth regulation, the underlying mechanisms by which they mediate the effects of biochar treatment remain largely unexplored. This study provides initial insights into key regulatory genes within *Kosteletzkya virginica* that are responsively upregulated following modified biochar application, thereby paving the way for subsequent mechanistic investigations into biochar-mediated stress amelioration.

## 4. Materials and Methods

### 4.1. Materials

The experimental soil was collected from a coastal saline–alkali area (34.77168072° N, 119.21959452° E) near Linhong River Estuary in Lianyungang New City, Jiangsu Province, China. Soil samples were taken from the 0~20 cm depth layer, air-dried naturally, ground, and passed through a 2-mm sieve for subsequent analysis. The physicochemical properties of the coastal saline–alkali soil were as follows: a pH of 8.95, electrical conductivity (EC) of 0.97 mS·cm^−1^, soluble salt content of 0.69%, and sodium adsorption ratio (SAR) of 130.69. The total nitrogen (TN), total phosphorus (TP), and total potassium (TK) contents were 1.13, 0.77, and 19.11 g·kg^−1^, respectively. The concentrations of available nitrogen (AN), available phosphorus (AP), and available potassium (AK) were 56.70, 8.26, and 475 mg·kg^−1^, respectively. The soil organic matter (SOM) content was 15.97 g·kg^−1^. The water-soluble K^+^, Ca^2+^, Na^+^, and Mg^2+^ contents were 60.67, 19.40, 726.50, and 11.50 mg·kg^−1^, respectively.

Seeds of *K. virginica* were provided by Jiangsu Lianyungang Golden Coast Development and Construction Co., Ltd., Lianyungang, China.

### 4.2. Biochar Preparation and Modification

Modified biochar composites were prepared using a phosphoric acid/mineral/biochar ratio (v:w:w) of 7.5:2:1 following Dai et al. [55]. Two co-modified biochar composites were prepared: HBCK (H_3_PO_4_–kaolinite-modified) and HBCM (H_3_PO_4_–montmorillonite-modified). Additionally, three single-modified biochar composites were produced: BCK (kaolinite-modified), BCM (montmorillonite-modified), and HBC (H_3_PO_4_-modified). The raw corn straw biochar (BC) served as a control.

### 4.3. Pot Experiment

The pot experiment included seven treatments: CK, BC, HBC, BCM, HBCM, BCK, and HBCK. All biochars were applied at 5% (*w*/*w*) with five replicates. Plants were grown in plastic pots (15 × 15 cm) containing 1.5 kg of soil mixed with the respective biochar treatments and 3.0% organic fertilizer. Five seeds of *K. virginica* were sown in each pot on 14 May 2023, with one seedling being retained per pot after germination. The experiment was conducted in a greenhouse with soil moisture maintained at >60% of field capacity. Samples were collected after 60 days of growth. The pH of the water used to maintain soil moisture during the vegetation period was 6.55.

### 4.4. Determination of Growth Parameters, Nutrient Contents, and Antioxidant Enzyme Activities in K. virginica

Plant height was measured pre-harvest using a measuring tape. At harvest, plants were separated into roots and shoots, washed, and weighed to determine fresh biomass. Shoot samples were oven-dried to a constant weight and then digested for subsequent analysis. Nitrogen content was determined by the Kjeldahl method using 5.00 mL aliquots of digestate. Phosphorus was measured colorimetrically at 880 nm after reaction with molybdenum–antimony reagent for 30 min. Potassium was analyzed directly using flame photometry following appropriate dilution. Root samples were ground in liquid nitrogen for enzyme assays. Superoxide dismutase (SOD) and peroxidase (POD) activities were determined using commercial kits (BC5165 and BC5160, respectively; Solarbio, Beijing, China) according to the manufacturer’s instructions.

### 4.5. RNA Extraction and Transcriptome Sequencing of K. virginica Roots

Total RNA was extracted from the roots of *K. virginica* subjected to CK, BC, HBC, and HBCK treatments using the TianGen RNA extraction kit (Beijing, China). RNA quality was assessed by measuring concentration and purity (OD260/280: 1.8–2.2) with a NanoDrop 2000 (Thermo Fisher Scientific, Wilmington, DE, USA), while integrity was verified via agarose gel electrophoresis. The RNA Quality Number (RQN > 6.5) was determined using an Agilent 5300 system. Only samples meeting the criteria (total RNA ≥ 1 μg, concentration ≥ 30 ng/μL) were used for library construction.

For transcriptome sequencing, eukaryotic mRNA was enriched via Oligo(dT) magnetic beads targeting the polyA tail. The mRNA was then fragmented into ~300 bp segments using a fragmentation buffer. First-strand cDNA was synthesized using random primers and reverse transcriptase, followed by second-strand synthesis to generate double-stranded cDNA. The cDNA was end-repaired, adenylated at the 3′ end, and ligated with sequencing adapters. After purification and size selection, the adapter-ligated fragments were PCR-amplified to construct the final library. High-throughput paired-end sequencing (150 bp) was performed on the NovaSeq X Plus platform.

### 4.6. Functional Annotation and Enrichment Analysis of Differentially Expressed Genes (DEGs)

Gene Ontology (GO) analysis was performed to categorize DEGs into three functional groups: biological processes (BPs), molecular functions (MFs), and cellular components (CCs) using the GO database (http://www.geneontology.org, accessed on 4 March 2025). Kyoto Encyclopedia of Genes and Genomes (KEGG) pathway annotation was conducted by mapping DEGs to the KEGG database (http://www.genome.jp/kegg, accessed on 7 March 2025) to obtain KO identifiers, which revealed potential biological pathways that were involved. Pathway enrichment significance was determined using hypergeometric tests with Benjamini–Hochberg correction, considering pathways with a false discovery rate (FDR) < 0.05 as significantly enriched. 

### 4.7. qRT-PCR Analysis

To ensure consistency, the same samples used for transcriptome sequencing were employed for qRT-PCR analysis. Gene-specific primers were designed using the Primer premier 5.0 (primer sequences provided in Supplementary Table S2). Reverse transcription was performed with 2 μg of root total RNA from different treatment groups of *K. virginica* using the HiScript II One-Step RT-PCR Kit (Vazyme Biotech, Nanjing, China) following the manufacturer’s protocol. qPCR amplification was conducted using Vazyme Taq Pro Universal SYBR qPCR Master Mix (Vazyme Biotech) on a Rotor-Gene 3000 Real-Time PCR system (Qiagen, Hilden, Germany). Primer specificity was verified using melting curve analysis. Relative gene expression levels were calculated using the 2^−ΔΔCT^ method [56]. Three biological replicates were included for each treatment.

### 4.8. Statistical Analysis

Data were analyzed using SPSS 20.0 (SPSS Inc., Chicago, IL, USA) and visualized with GraphPad Prism 8 (GraphPad Software, San Diego, CA, USA). Prior to parametric testing, normality (Shapiro–Wilk test) and homogeneity of variance (Levene’s test) were assessed. For normally distributed data with equal variances, treatment differences were evaluated using one-way ANOVA followed by Duncan’s post hoc test, with significant differences indicated by lowercase letters. Non-normal data were analyzed using the Kruskal–Wallis test.

## 5. Conclusions

Building upon a series of successfully prepared modified biochars, this research systematically evaluated their regulatory effects on the halophyte *K. virginica* in saline soil. Cultivation experiments demonstrated that the HBC and HBCK treatments significantly outperformed the others, markedly enhancing plant height and biomass. Multidimensional analyses revealed the following underlying mechanisms: (1) enhanced nutrient uptake (N, P, K); (2) elevated antioxidant enzyme activity (SOD, POD), which mitigated ROS accumulation; (3) transcriptome analysis indicated the activation of key transcription factors (HSP, MYB, AP2/ERF) and regulation of ABA/JA signaling genes (e.g., CYP707A, OPR2, LOX2), forming an integrated “nutrient–antioxidant–molecular” network that coordinately enhances salt tolerance and growth. This work clarifies the multidimensional mechanisms underpinning modified biochar efficacy in saline phytoremediation. However, the functional validation of key regulatory genes and full elucidation of molecular mechanisms are pending. This study’s scope was limited to a single species without long-term amendment assessment. Future work will employ functional genomics to validate key genes for breeding applications and evaluate the growth-promoting effects and long-term performance of these modified biochars across diverse halophytes. This will assess universal applicability and identify synergistic biochar–plant combinations for targeted saline–alkali soil remediation.

Subsequent functional validation of these key genes will provide molecular targets for breeding programs aimed at vegetation restoration in saline–alkali soils.

## Figures and Tables

**Table 1:** Statistical results of transcriptome sequencing.

Sample	Total Reads	Mapped Reads	GC Content	Q20 (%)	Q30 (%)
CK-1	25,686,502	22,626,954 (88.09%)	45.3	98.8	96.34
CK-2	25,734,167	22,678,505 (88.13%)	45.08	98.8	96.35
CK-3	24,271,463	21,317,319 (87.83%)	44.99	98.76	96.25
BC-1	22,125,697	19,337,469 (87.40%)	45.13	98.74	96.13
BC-2	24,332,344	21,323,199 (87.63%)	45.36	98.75	96.18
BC-3	23,895,927	21,033,680 (88.02%)	45.09	98.77	96.24
HBC-1	21,542,792	18,929,233 (87.87%)	45.2	98.7	95.99
HBC-2	20,394,966	17,864,767 (87.59%)	45.1	98.73	96.12
HBC-3	22,663,117	19,920,356 (87.90%)	45.14	98.71	96.07
HBCK-1	22,558,390	19,912,614 (88.27%)	45.24	98.75	96.16
HBCK-2	21,589,175	19,084,337 (88.40%)	45.11	98.75	96.2
HBCK-3	26,135,691	23,134,737 (88.52%)	45.26	98.79	96.31

Note: CK: control group; BC: raw corn straw biochar; HBC: H_3_PO_4_-modified biochar; HBCK: H_3_PO_4_–kaolinite–biochar composite.

**Table 2:** Gene function annotations.

Annotated Database	Annotated Number	Annotated Percent (%)
GO	37,354	50.96
KEGG	18,529	25.28
eggNOG	34,955	47.69
NR	43,828	59.79
Swiss-Prot	32,555	44.41
Pfam	28,317	38.63
Total_annotated	44,137	60.21

Note: GO: Gene Ontology; KEGG: Kyoto Encyclopedia of Genes and Genomes; eggNOG: Evolutionary Genealogy of Genes: Non-Supervised Orthologous Groups; NR: Non-Redundant Protein Database; Pfam: Protein Family.

## Data Availability

The original data presented in the study are openly available in China National Center for Bioinformation at CRA025851.

## Supplementary Materials

The following supporting information can be downloaded at: https://www.mdpi.com/article/10.3390/plants14121849/s1, Table S1: RNA extraction concentration analysis of *K. virginica* roots; Table S2: Real-time PCR primers for internal reference and differentially expressed genes; Figure S1: RNA gel electrophoresis (A) and sequence length distribution of transcripts (B).
