# Supplementary material for: Effects of Different Modified Biochars on Growth of Kosteletzkya virginica and Corresponding Transcriptome Analysis
Source: Plants (Basel). 2025 Jun 16;14(12):1849. doi: 10.3390/plants14121849 (PMC12196827; doi:10.3390/plants14121849)
Supplement: Supplementary file 1 [file plants-14-01849-s001.zip › plants-3647944-supplementary.pdf]

Article

Effects of Different Modified Biochars on the Growth of *Kosteletzkya virginica* and Corresponding Transcriptome Analysis

Hao Dai [1,3], Mingyun Jia [1], Jianhui Xue [1,3], Yuying Huang [2,*] and Jinping Yu [1,*]

1 Jiangsu Key Laboratory for the Research and Utilization of Plant Resources, Institute of Botany, Jiangsu Province and Chinese Academy of Sciences (Nanjing Botanical Garden Mem. Sun Yat-Sen), Nanjing 210014, China; hdai@njfu.edu.cn (H.D.); jiamingyun@jib.ac.cn (M.J.); jhxue@njfu.edu.cn (J.X.);

2 College of Biomedicine and Health, Anhui Science and Technology University, Chuzhou 233100, China

3 College of Ecology and Environment, Nanjing Forestry University, Nanjing 210037, China

* Correspondence: yujinping@cnbg.net (J.Y.); 13202078270@163.com（Y.H.)

Table S1 RNA extraction concentration analysis of K. virginica root

Table S2. Real-time PCR primers for internal reference and differentially expressed genes

Figure S1 RNA gel electrophoresis (A) and sequence length distribution of transcript (B)

Table S1. RNA extraction concentration analysis of *K. virginica* root

Sample	RNA concentration（ng/μL）	A260/A280
CK-1	723.8	2.15
CK-2	406.1	2.13
CK-3	606.7	2.15
BC-1	542.5	2.16
BC-2	445.8	2.11
BC-3	439.3	2.12
HBC-1	507.3	2.14
HBC-2	397.4	2.11
HBC-3	874.1	2.16
HBCK-1	456.3	2.13
HBCK-2	401.9	2.11
HBCK-3	312.8	2.11

Table S2. Real-time PCR primers for internal reference and differentially expressed genes.

Primer Name	Primer Sequence （5’-3’）
qKpActin-F	TCCCTCAGCACATTCCAGCAGAT
qKpActin-R	AACGATTCCTGGACCTGCCTCATC
qKpCKX-F	GAACTGGCTTTCCATTATCG
qKpCKX-R	GCACATCTACCTGGGATTTC
qKpPOD72-F	AGCGGGACTACAATAAGCG
qKpPOD72-R	GAACAGTTGAATCTCTGGCAG
qKpCYP90-F	CAACTTTACCGTTCCTCGC
qKpCYP90-R	CACTTCACTCGCATCCTTG
qKpOPR2-F	CGATGAGGAAAGCCTTCAAG
qKpOPR2-R	CGAATGCGACCAAATCTG
qKpNQO1-F	CGCTTTGGAGATTAGTAAGGAG
qKpNQO1-R	GGAAACTTGCCATCAACTTC
qKpHSFA2-F	CAGATAGATGGGAGTTTGCC
qKpHSFA2-R	TCCACCTTGATGTTGTGAAC
qKpTIE-F	CGGCAATGGCATCTTAGAC
qKpTIE-R	GCTCCTGTGTGTCACTTGATAC
qKpNLP-F	GGTCTGTCATCAAATCCAGC
qKpNLP-R	CACACAAGGCAGTGAAACC
qKpMYB108-F	ATTGCGGGAATGGTATCC
qKpMYB108-R	GCTGCTGCTGTAATAATCGG

A

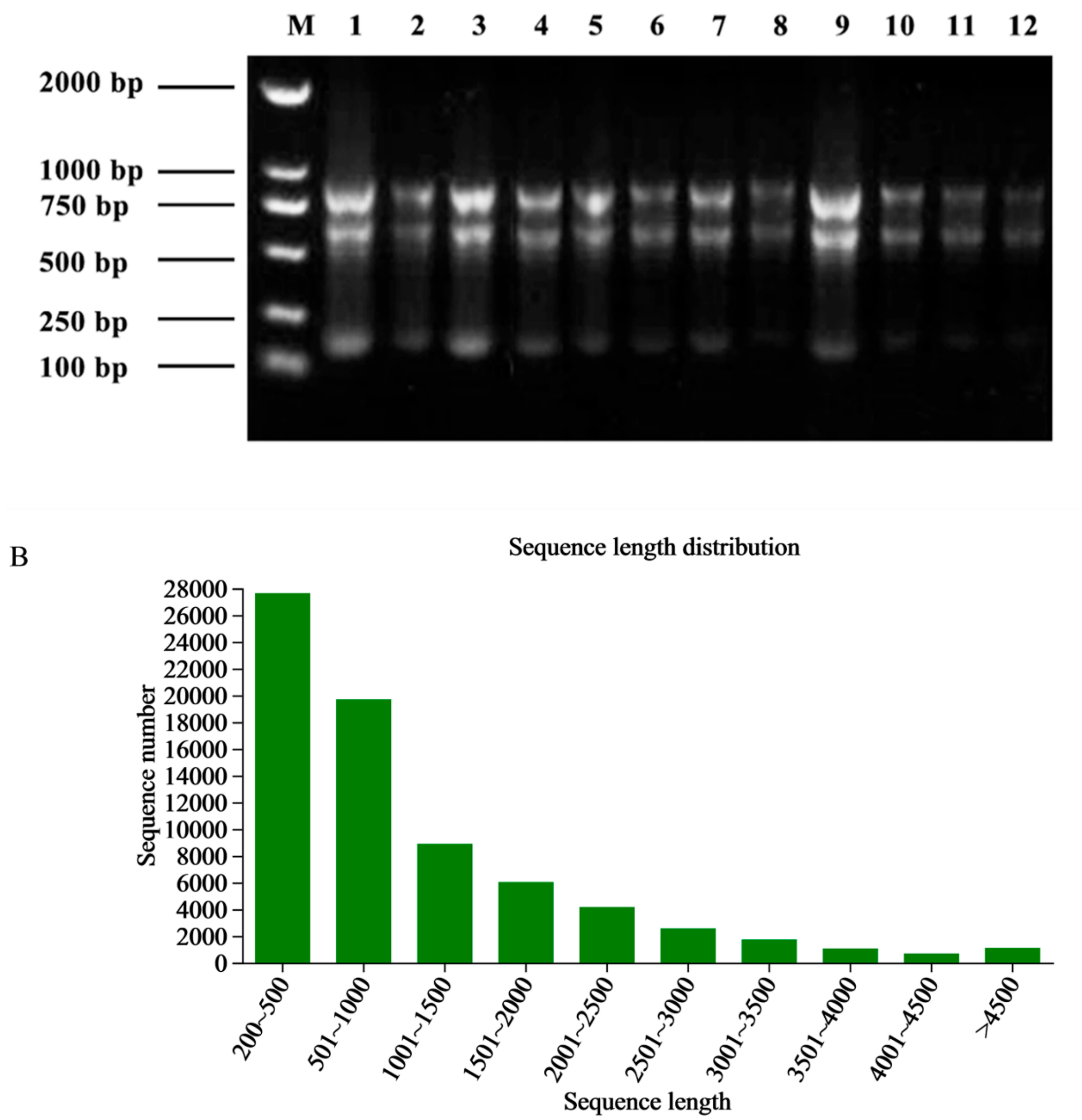

Figure S1. RNA gel electrophoresis (A) and sequence length distribution of transcript (B).
